# Bacterial microbiome of root-associated endophytes of *Salicornia europaea* in correspondence to different levels of salinity

**DOI:** 10.1007/s11356-018-2530-0

**Published:** 2018-06-27

**Authors:** Sonia Szymańska, Luigimaria Borruso, Lorenzo Brusetti, Piotr Hulisz, Bliss Furtado, Katarzyna Hrynkiewicz

**Affiliations:** 1 Department of Microbiology, Faculty of Biology and Environmental Protection, N. Copernicus University in Torun, Lwowska 1, Toruń, Poland; 2 Faculty of Science and Technology, Free University of Bozen – Bolzano, Piazza Università 5, 39100 Bolzano, Italy; 3 Department of Soil Science and Landscape Management, Faculty of Earth Sciences, N. Copernicus University in Torun, Lwowska 1, Toruń, Poland

**Keywords:** Soil salinity, Endophytes, Bacteria, *S. europaea*, Microbiome, Halophyte

## Abstract

The halophytes have evolved several strategies to survive in saline environments; however, an additional support from their associated microbiota helps combat adverse conditions. Hence, our driving interests to investigate the endophytic bacterial community richness, diversity, and composition associated to roots of *Salicornia europaea* from two test sites with different origins of soil salinity. We assumed that salinity will have a negative effect on the diversity of endophytes but simultaneously will permit the high occurrence of halophylic bacteria. Further, to establish the role of the host and its external environment in determining the endophytic diversity, we analyzed the physico-chemical parameters of root zone soil and the concentration of salt ions in the plant roots. The results based on the Miseq Illumina sequencing approach revealed a higher number of endophytic bacterial OTUs at naturally saline test site with a higher level of soil salinity. Proteobacteria and Bacteriodetes were the dominant endophytic phyla at both analyzed sites; additionally, the high occurrence of Planctomycetes and Acidobacteria at more saline site and the occurrence of Firmicutes, Actinobacteria, and Chloroflexi at less saline site were recorded. The salinity in the root zone soil was crucial in structuring the endophytic community of *S. europaea*, and the significant prevalence of representatives from the phyla Deltaproteobacteria, Acidobacteria, Caldithrix, Fibrobacteres, and Verrucomicrobia at the more saline test site suggest domination of halophylic bacteria with potential role in mitigation of salt stress of halophytes.

**Electronic supplementary material:**

The online version of this article (10.1007/s11356-018-2530-0) contains supplementary material, which is available to authorized users.

## Introduction

Halophytes are a small group of plants (approx. 1%), which are able to grow and develop in soils with high levels of salinity (from 200 mM NaCl) (Flowers and Colmer Flowers and Colmer 2008). The mechanisms responsible for the high tolerance of halophytes to saline environments include ion compartmentalization, osmotic adjustment, succulence, ion transport and uptake, antioxidant systems, maintenance of redox and energetic status, and salt inclusion/excretion (Lokhande and Suprasanna 2012). *Salicornia europaea* (*herbacea*) L. (Amaranthaceae) is one of the most salt-tolerant halophyte plant species in the world (tolerating more than 1000 mM NaCl) (Lv et al. 2012; Feng et al. 2015). The mechanisms of salt tolerance in *S. europaea* have been assessed using physiological, proteomic and transcriptomic approaches (Wang et al. 2009; Fan et al. 2011; Lv et al. 2012; Fan et al. 2013; Ma et al. 2013). *S. europaea* is considered to be an appropriate model plant to study response to salt stress and efficiency of desalination processes (e.g., Lokhande and Suprasanna 2012; Lv et al. 2012; Rozema and Schat 2013; Feng et al. 2015). This halophyte occurs in Europe, South Africa, South Asia, and North America, especially near coastlines, tidal floodways, and salt lakes (Rhee et al. 2009; Jafari et al. 2012). Moreover, its therapeutic applications, including antioxidant, antidiabetic, anticancer, hypocholesterolemic, and anti-aging properties (Jung et al. 2008; Lee et al. 2012), have been widely studied. Due to the wealth of chemical constituents (e.g., sterols, caffeoylquinic acid derivatives, flavonoid derivatives, triterpenoid saponins, pentadecylferulate) and its dietary fiber content, it is an attractive plant suitable for use in the treatment of obesity, diabetes, asthma, arthritis, and constipation (Ha et al. 2006; Cho et al. 2016).

Since the growth and development of all living plants are associated with their microbial counterpart, it may be suggested that the mechanisms for adaptation of halophytes to high salinity are possibly connected with highly specialized, halotolerant endophytic and rhizospheric microorganisms. These microorganisms may facilitate plant growth and tolerance to adverse environmental conditions (Sgroy et al. 2009; Arora et al. 2012; Berg et al. 2013; Szymańska et al. 2016a, 2016b; Borruso et al. 2014).

Endophytes are bacteria and fungi that colonize plant tissues, without causing symptoms of disease in their host (Glick 2012; Eljounaidi et al. 2016). The close interaction of endophytes within the host plant can directly affect plant properties, as compared to the interaction of the rhizosphere bacteria (Compant et al. 2010; Rashid et al. 2012; Eljounaidi et al. 2016). The main source of endophytes is the rhizosphere, and rarely phyllosphere (aboveground part of the plant colonized by microorganisms), but only a negligible part of them has the ability to spread through the seeds (particularly obligatory endophytes) (Ryan et al. 2008; Gaiero et al. 2013). Endophytes diversity can be closely related to plant physiology and environmental conditions (Miliute et al. 2015). However, it has mostly been evaluated using culture-dependent techniques (Shi et al. 2015; Szymańska et al. 2016a), and several reports have already confirmed plant growth promoting properties of endophytic bacteria associated with halophytic plants (Sgroy et al. 2009; Jha et al. 2012; Kannan et al. 2014; El-Awady et al. 2015). Unfortunately, culture-dependent approaches illustrate only about 0.01–1% of the microbial species present in the environment such as soil, seawater, and marine sediments (Garza and Dutilh 2015). As a result, there is a strong need for highly sophisticated methods to assess the diversity of endophytic bacteria in halophyte plants. Culture-independent approaches have been employed to evaluate the population structure of endophytes associated with halophytes, even with respect to *S. europaea* (Shi et al. 2015; Zhao et al. 2016); however, detailed reports indicating a relationship between biodiversity of root endophytes and broad studies of physico-chemical parameters of soils, and the level of salinity are meager. Moreover, the comparisons of different techniques (e.g., identification and metabolic characterization of different isolates, Biolog phenotype microarray technique, phospholipid fatty acids analysis (PLFA)) used for analysis of endophytes of *S. europaea* in our previous work (Szymańska et al. 2014; Szymańska et al. 2016a) along with the present study in advanced metagenomic analysis are very important. We intend to demonstrate our research work using a multifaceted approach to taxonomic structure and metabolic potential as well as from a methodological viewpoint and compare results from these different techniques. Understanding the taxonomic structure and exploring the biodiversity of endophytic bacteria associated with *S. europaea* in relation to physico-chemical parameters of root zone soil can explain the complex and intimate relationships that exist between microorganisms and plants growing in saline areas.

The aim of our study was to assess the taxonomic diversity of endophytic bacterial community associated with the roots of obligatory halophytic plant *S. europaea* L. growing at two saline test sites in relation to the physico-chemical parameters of root zone soil using metagenomic techniques based on 16S rRNA. We hypothesized that (i) higher levels of soil salinity decrease density and increase the microbial diversity of more specialized halophilic endophytic bacteria, and (ii) the interaction between the host plant and its environment play a role in shaping the bacterial endophytic community in *S. europaea* and favoring the occurrence of halophilic strains.

## Materials and methods

### Site description and plant sampling procedure

The two saline test sites (C and I) are located in the central part of Poland (at a distance not exceeding 37 km) and characterized by natural (C) and anthropogenic (I) origin of salinity. The site (C) is located in Ciechocinek (52°53 N, 18°47 E) and comprises of 1.88 ha of the Halophyte Nature Reserve that was established in 1963 (included in the Natura 2000 network in 2008). The sampling area is situated close to a brine graduation tower built in the middle of the nineteenth century. The soil salinization at site C is associated with the Zechstein salt deposits, which were uplifted to the surface in this area and have been exploited since the Middle Ages. Site C is the only natural inland site in Poland where *S. europaea* and *Aster tripolium* halophytes occur (Piernik 2006). The site I comprises of meadows near a soda factory (Soda Poland CIECH SA, founded in 1879) (Inowrocław, 52°48 N, 18°15 E). Because of inappropriate waste storage from soda ash production (Solvay’s method), it has led to alkalization and salinization of soils (Hulisz et al. 2017). Site I is characterized by wide range of the soil salinity, which has favoured the occurrence of different species of halophytes, e.g., *S. europaea*, *Aster tripolium*, *Spergularia salina*, *Glaux maritima*, *Triglochin maritimum*, *Puccinellia distans*, and *Atriplex prostrata* var. *salina*, as well as species less resistant to salt stress like *Bolboschoenus maritimus*, *Lotus tenuis*, *Tetragonolobus maritimus*, *Festuca rubra*, and *Trifolium fragiferum* (Piernik et al. 2015).

At each test site, nine samples (three each from three sub-plots or 3 × 3 sub-plots) of *S. europaea* with the soils adjacent to the roots (20 cm × 20 cm, 20-cm soil cubes) were collected in autumn 2015 using sterile tools. In order to avoid contamination, the samples were placed in separate sampling bags and immediately transported to the laboratory for analysis.

### Root zone soil description

The root zone soil samples were gently separated from roots and analyzed according to the methodology described by Hrynkiewicz et al. (2010). The air-dried samples were sieved (2-mm mesh) and analyzed according to the following methods: total organic carbon (OC) and total nitrogen (N_t_) content using a CNS Variomax analyzer, and CaCO_3_ content by Scheibler volumetric method (Bednarek et al. 2004).

The saturation paste extracts were prepared to evaluate the soil salinity level. The electrical conductivity (EC_e_) was measured by the conductometric method, pH_e_ by the potentiometric method, and saturation percentage (SP)—gravimetrically (van Reeuwijk 2002). Moreover, the content of ions in extracts was determined: Na^+^, K^+^, Ca^2+^, and Mg^2+^ by the AAS method, Cl^−^ argentometrically, SO_4_^2−^ nephelometrically, and HCO_3_^−^ acidimetrically.

*T*hree replicates of plant samples were taken, mixed, and prepared for chemical analysis in the following stages: drying at 60 °C, homogenization, dry combustion at 460 °C, and hot mineralization using the mixture of HNO_3_ and H_2_O_2_ (Piper 1966; Czerniawska-Kusza et al. 2004, with modification). The total content of calcium, magnesium, sodium, potassium, and chlorides was determined in the obtained solutions in accordance with the methods used for soil extracts.

### Plant surface sterilization, DNA extraction, and PCR amplification

Roots of *S. europaea* from each sample were separated from the adhering soil, washed with 2% NaCl, dried using sterile filter paper, and weighed to establish biomass. The surface sterilization of roots was carried out using 70% ethanol (2 min), followed by washing with sterile 2% NaCl (three times), sterilized with 15% of H_2_O_2_ (5 min), and finally washed with sterile 2% NaCl (three times). The solutions obtained after the final washing (for each analyzed sample, 18 in total) were evaluated for surface sterilization efficiency by plating on agar and monitored for microbial growth. Only successfully sterilized root material was used for further analysis. From each sample (18 in total), 1 g of fresh biomass of sterile roots was stored in 2-ml Eppendorf tubes and lyophilized.

Isolation of DNA from 20 mg of freeze-dried root material was performed according to the protocol (Plant and Fungi DNA purification kit, EURx). The isolated DNA was placed in 1.5-ml Eppendorf tubes and was send in cold packs (Blue ice) to IGA Technology Services (Udine, Italy) for analysis of amplicons (Illumina Platform—MiSeq).

### Illumina sequencing data analysis of bacterial 16S rRNA genes

The region of V3-V4 16S rRNA was amplified using primers S-D-Bact-0341-b-S-17 (5′- CCT ACG GGN GGC WGC AG - 3′) and S-D-Bact-0785-a-A-21 (5′- GAC TAC HVG GGT ATC TAA TCC - 3′) (Klindworth et al. 2013). The PCR products were sequenced using the Illumina MiSeq platform, as per the instructions provided in the manufacturer’s manual. Paired-end reads from each library were merged via Pear v.0.9.2 (Zang et al. 2014). The sequences were trimmed with a quality score threshold of − ≤ 30 and shorter than 250 bp were discarded. Assembled reads were analyzed using Qiime v.1.8 software package (Caporaso et al. 2010). Sequences were checked for chimeras with the Chimera VSEARCH (Rognes et al. 2016). Subsequently, the sequences were taxonomically annotated using GreenGenes database (http://greengenes.secondgenome.com/). Operational taxonomic unit (OTU) table was generated using demultiplexed sequences at 97% similarity, and singletons were removed. All samples have been submitted to the European Nucleotide Archive under accession numbers from ERS1981160 to ERS1981177.

### Statistical analysis

Determination of differences between two investigated sites (C and I) on the level of physico-chemical soil parameters was assessed by ANOVA with parametric Newman-Keuls test as a post hoc comparison using Statistica software (Statistica ver. 7, StatSoft). Venn diagram was generated using Bioinformatics and System Biology. PAST (Hammer et al. 2001) was used for non-metric multidimensional scaling (NMDS) by using Bray–Curtis dissimilarity distance, and the environmental variables were overlaid on the ordination plots. Linear discriminant analysis (LDA) effect size (LEfSe) was applied to identify indicator bacterial groups specialized within the two different sites (Segata et al. 2011).

## Results

### Root zone soil and roots of *S. europaea* parameters

The physico-chemical parameters of root zone soil of *S. europaea* growing at two saline sites (C and I) are presented in Table 1. Among the 14 studied soil parameters (OC, N_t_, CaCO_3,_ C:N, SP, pH_e_, EC_e_, Na^+^, K^+^, Ca^2+^, Mg^2+^, Cl^−^, SO_4_^2−^, HCO_3_^−^), 11 revealed significant differences between sites. In general, higher average percentages of organic carbon (OC), total nitrogen (N_t_), and Ca^2+^ (g dm^−3^) were recorded in the root zone soil of *S. europaea* from test site I characterized by lower salinity with anthropogenic origin. Higher levels of eight investigated parameters were noted for root zone soil at site C (Table 1). The level of C/N, pH_e_, and HCO_3_^−^ was similar at both test sites (Table 1). The total element content of *S. europaea* roots is presented in Table 2. In general, a significantly higher level of all tested root parameters (Ca, Mg, Na, K, and Cl) was noted for the roots of *S. europaea* collected from more saline site C (Table 2).

**Table 1:** Physico-chemical root zone soil parameters (mean of nine replicates and standard deviation) in autumn 2015

Parameter/site	C	I
OC (%)	3.81 (1.32)	5.88 (1.67) [↑]
N_t_ (%)	0.35 (0.08)	0.52 (0.14) [↑]
CaCO_3_ (%)	39.1 (5.63) [↑]	28.7 (12.4)
C/N	11 (2)	11 (3)
SP (%)	98.0 (13.7) [↑]	85.9 (8.00)
pH_e_	6.9 (0.1)	6.9 (0.1)
EC_e_ [dS m^−1^]	113 (8.72) [↑]	63.7 (9.37)
Na^+^ [g dm^−3^]	25.4 (2.46) [↑]	7.95 (0.89)
K^+^ [g dm^−3^]	0.50 (0.09) [↑]	0.12 (0.03)
Ca^2+^ [g dm^−3^]	2.37 (0.32)	9.88 (1.05) [↑]
Mg^2+^ [g dm^−3^]	0.59 (0.06) [↑]	0.16 (0.04)
Cl^−^ [g dm^−3^]	47.3 (4.91) [↑]	29.9 (2.94)
SO_4_^2−^ [g dm^−3^]	0.31 (0.02) [↑]	0.10 (0.02)
HCO_3_^−^ [g dm^−3^]	0.12 (0.04)	0.10 (0.04)

[↑] significantly higher level based on Newman-Keuls test of root zone soil parameter observed between the sites

**Table 2:** Physico-chemical *S. europaea* roots parameters (mean and standard deviation) in autumn 2015

Site/parameter	Na (mg kg^−1^)	K	Ca	Mg	Cl
C	6149 [↑] (518.0)	10585 [↑] (1444.0)	113910 [↑] (22320.0)	13922.5 [↑] (154.5)	6609.5 [↑] (2176.5)
I	2711.5 (377.5)	1780.5 (177.5)	58671 (6443.0)	9074 (1002.0)	1252 (477.0)

[↑] significantly higher level based on Newman-Keuls test of *S. europaea* roots parameter observed between two tested sites (I and C)

### Endophytic bacterial community structure and soil parameters

**Fig. 1:**
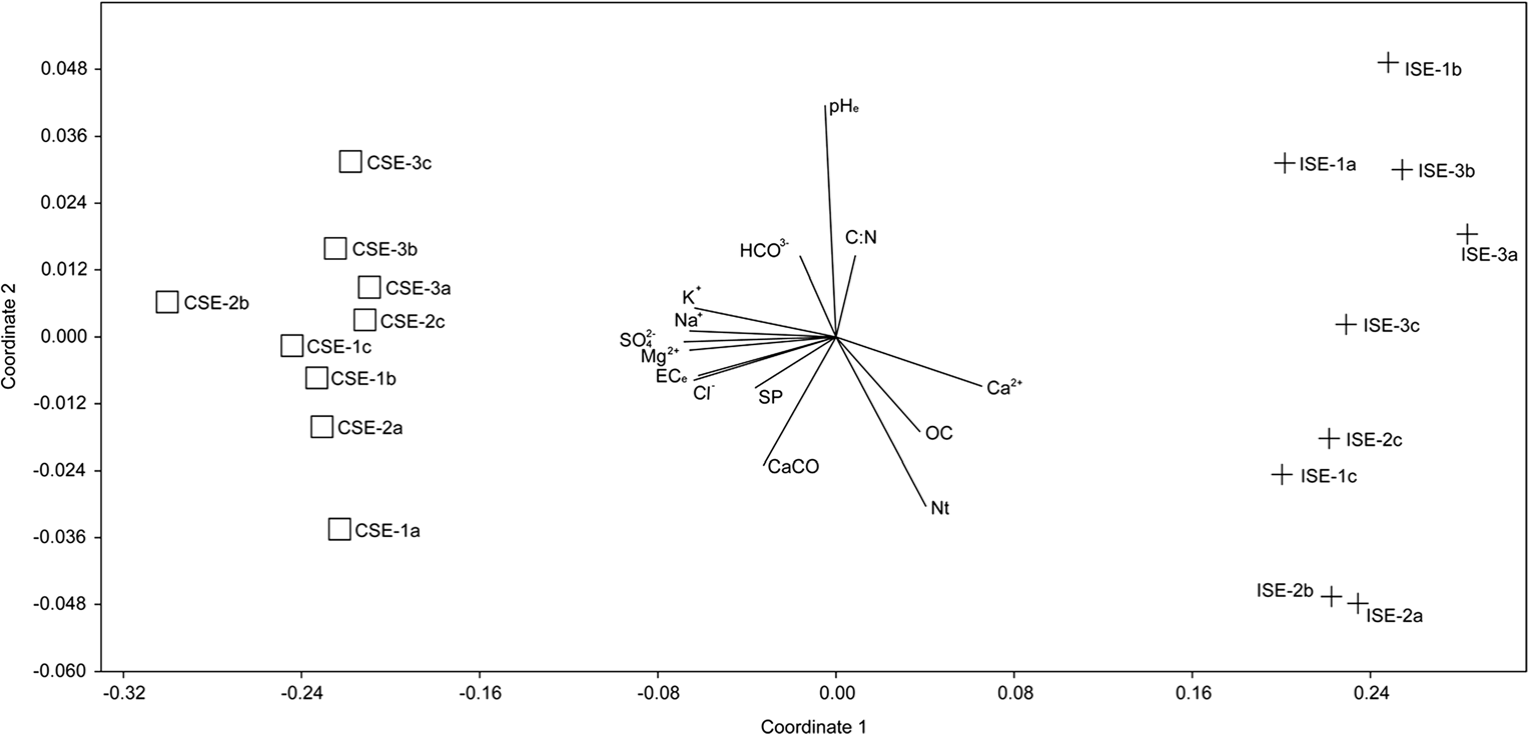
Non-metric multidimensional scaling, diagrams with axes 1 and 2 for endophytic bacteria associated with *S. europaea* roots (including diversity of OTUs) collected from site C (CSE) and site I (ISE) including 3 plots (1, 2, and 3) and three replications (a, b, and c) and for 14 chemical soil parameters (OC, EC_e_, *N*_*t*_, pH_e_, C/N, SP, CaCO_3_, Ca^2+^, K^+^, Na^+^, Mg^2+^, Cl^−^, SO_4_^2−^, HCO_3_^−^). *p ≤ 0.05, significant factors

The endophytic bacterial community structure and the influence of the soil physico-chemical parameters were investigated via non-metric multidimensional scaling (NMDS). The NMDS plot clearly separated the endophytic microbial communities inhabiting the two sites (C and I). NMDS goodness of fit of the stress value was 0.1. The coefficients of determination between distances along each axis and the original distance (*R*^2^) were 0.91 and 0.02 for axis 1 and 2, respectively (Fig. 1). The endophytic bacterial communities from site C were positively correlated with the majority of the tested root zone soil parameters: CaCO_3,_ SP, EC_e_, Na^+^, K^+^, Mg^2+^, Cl^−^, SO_4_^2−^, HCO_3_^−^, while endophytic bacterial community from site I was closely related to OC, N_t_, Ca^2+^, and C/N (Fig. 1).

### Endophytic bacterial community composition and diversity

**Fig. 2:**
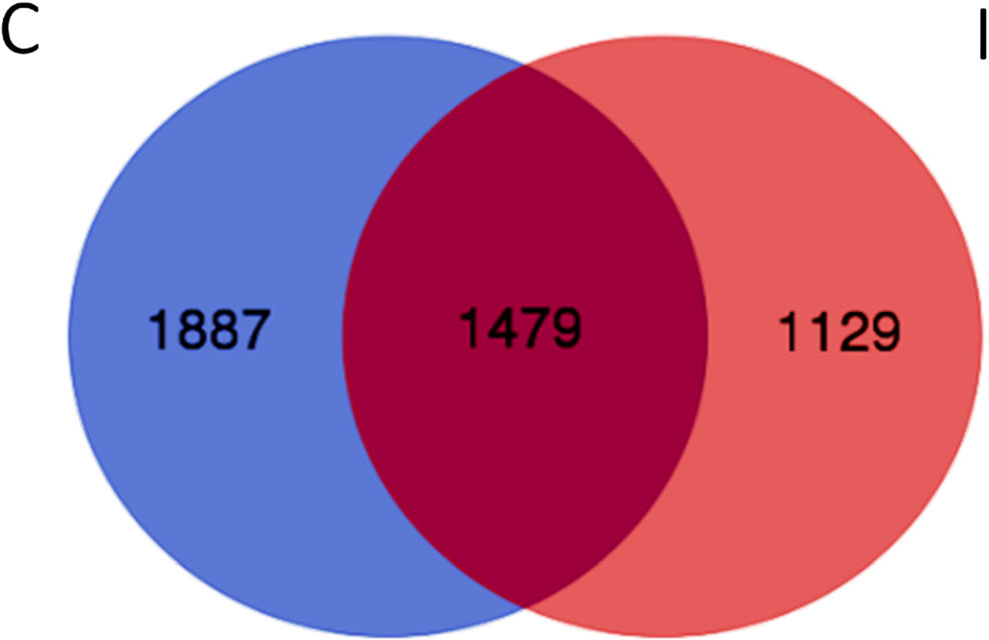
Venn diagram presenting the amount of OUTs for *S. europaea* roots endophytes from site C (1887) and site I (1129), 1479 common

The yield of MiSeq Illumina run after quality check and removal of singletons gave 186,632 sequences from all the samples. The total number of OTUs was 4495, which are presented as a Venn diagram in Fig. 2. The results show a higher number of OTUs identified for samples collected from the more saline site C (3366) as compared to the number of OTUs found in samples from the less saline site I (2608). Moreover, 1479 common OTUs were identified for the bacterial community in both the groups (Fig. 2). We have not observed statistically significant differences in the species diversity of endophytic bacteria between the investigated sites (Table 3).

**Table 3:** Comparison of the estimated operational taxonomic unit (OTU) richness and the diversity indices of the 16S rRNA gene libraries obtained from the pyrosequencing analysis

Site/diversity index	OTUs richness	Shannon_H index	Evenness	Chao 1 index
CSR	1160.22 (370.731) a	4.83 (0.311) a	0.12 (0.042) a	1715.11 (364.704) a
ISR	759.89 (248.028) a	5.30 (0.237) a	0.27 (0.025) a	1143.18 (275.249) a

Mean values are presented (*n* = 9). Letters in a single line indicate significant (*p* ≤ 0.05) differences one-way ANOVA with Newman-Keuls post hoc comparison among the diversity index

**Fig. 3:**
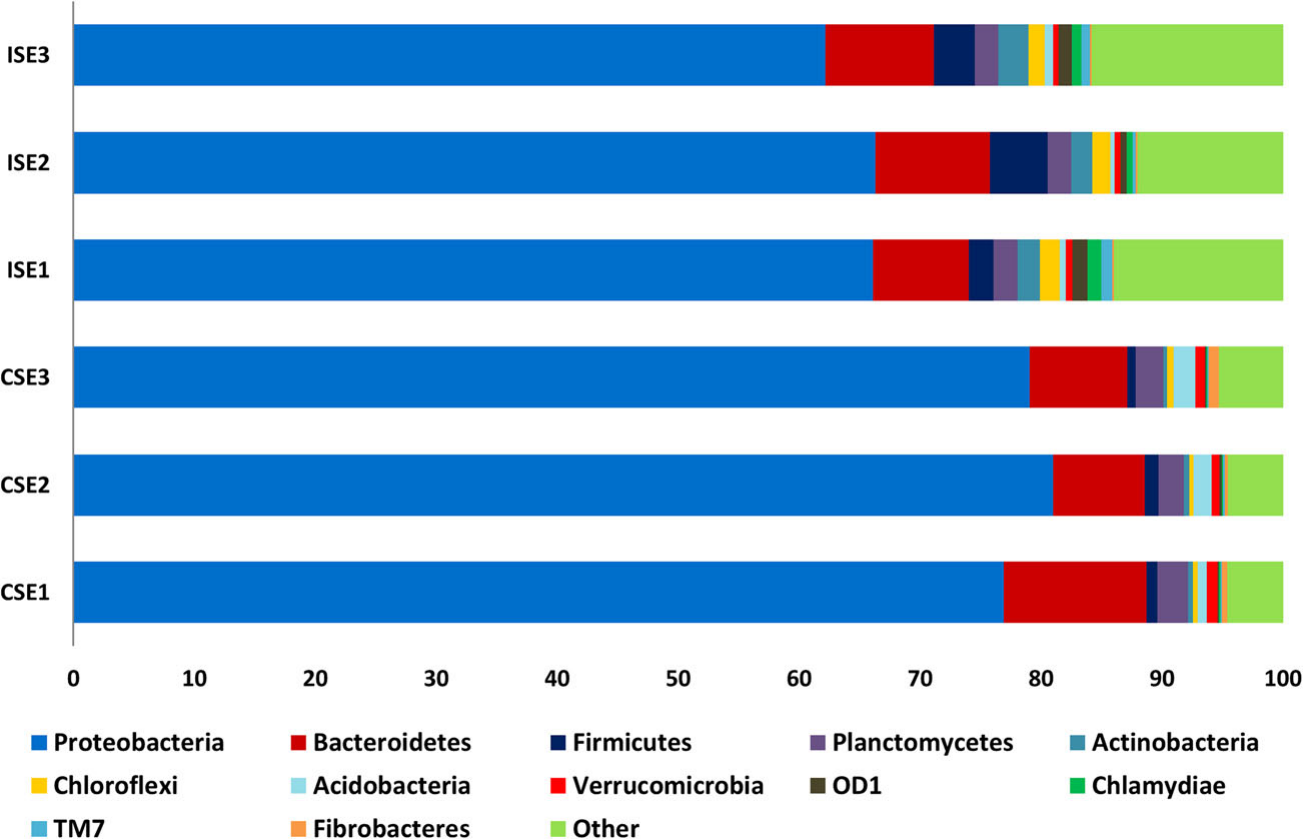
Comparison of bacterial communities from interior of roots of *S. europaea* collected from two saline sites (C and I) at the phylum level. Phyla abundances lower than 5% were shown as “other”

**Fig. 4:**
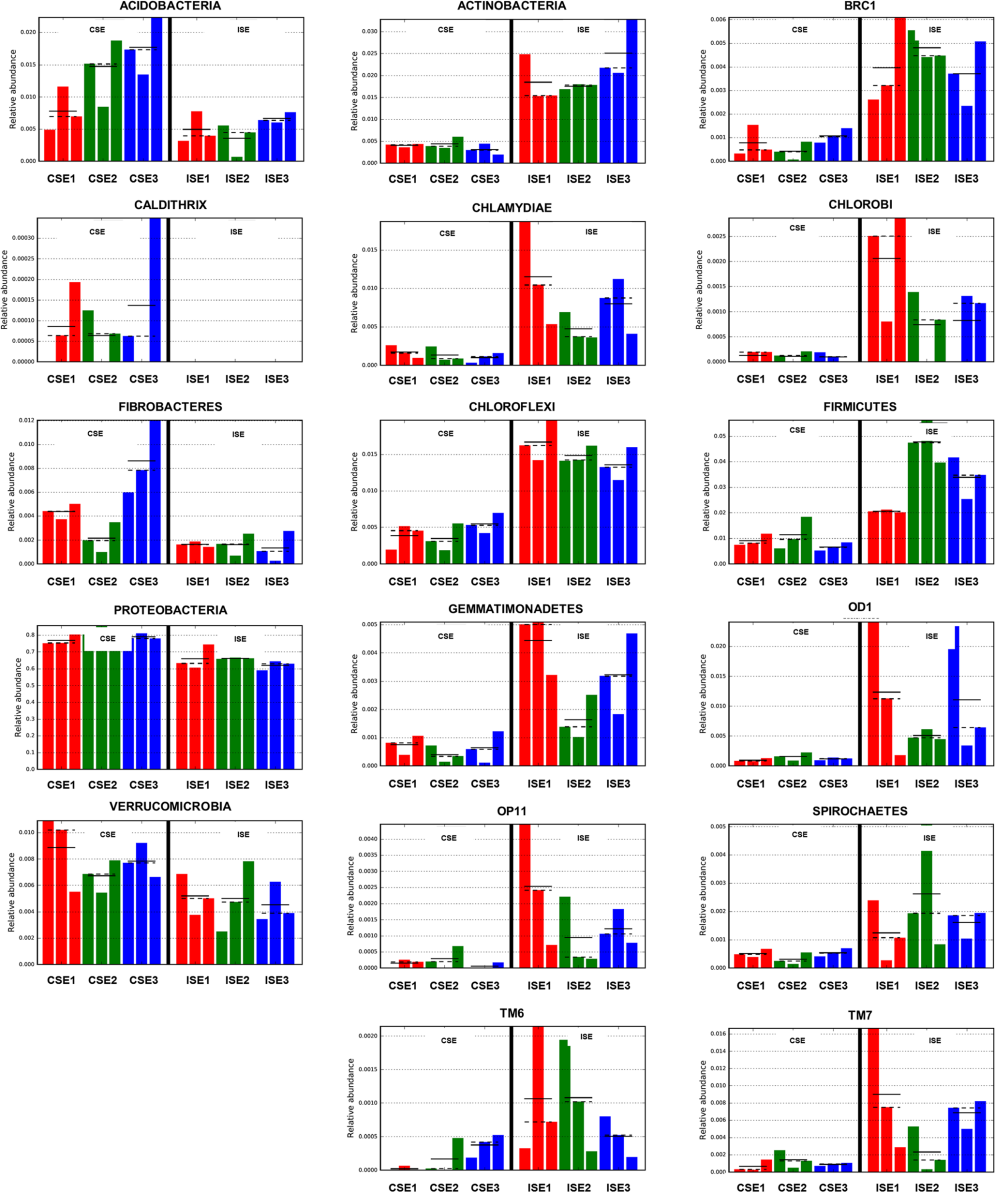
Bacterial taxa significantly differentiated between the higher, natural salinity site C (CSE) and less, anthropogenic salinity site I (ISE) identified using linear discriminant analysis coupled with effect size (LEfSe) based on relative abundance

The analysis of all sequences revealed the presence of 36 phyla, among which 12 phyla (Proteobacteria, Bacteriodetes, Firmicutes, Planctomycetes, Actinobacteria, Chloroflexi, Acidobacteria, Verrucomicrobia, OD1, Chlamydiae, TM7, Fibrobacteres) was seen to dominate among the endophytes from both the test sites (Fig. 3). The pattern of distribution of each phylum was associated with the site of isolation. Phylum Proteobacteria was the most dominant at both test sites: C (exceeds 78%) and I (above 64%). Bacteriodetes was observed at similar frequencies at sites C and I (about 9%). Firmicutes, Actinobacteria, and Chloroflexi were noted frequently at site I (3.4, 2.0, 1.5%, respectively) as compared to C (0.9, 0.4, 0.4%, properly), while Planctomycetes and Acidobacteria were identified repeatedly among bacteria from C (2.3% and 1.3%) than from I (1.9 and 0.5%) (Fig. 3). Unassigned and the rest of identified phyla were presented as other (Fig. 3). Linear discriminant analysis coupled with effect size (LEfSe) based on relative abundance of bacterial core OTUs demonstrated 17 phyla with a significant effect on two saline sites (Fig. 4). The endophytic bacteria such as Acidobacteria, Caldithrix, Fibrobacteres, Proteobacteria, and Verrucomicrobia were observed to be mostly associated with *S. europaea* originating from the more saline site (C) than less saline site (I) (Fig. 4). The opposite trend was observed for Actinobacteria, BRC1, Chlamydiae, Chlorobi, Chloroflexi, Firmicutes, Gemmatimonadetes, OD1, OP11, Spirochates, TM6, and TM7 whose abundance was higher at the site I (Fig. 4).

**Fig. 5:**
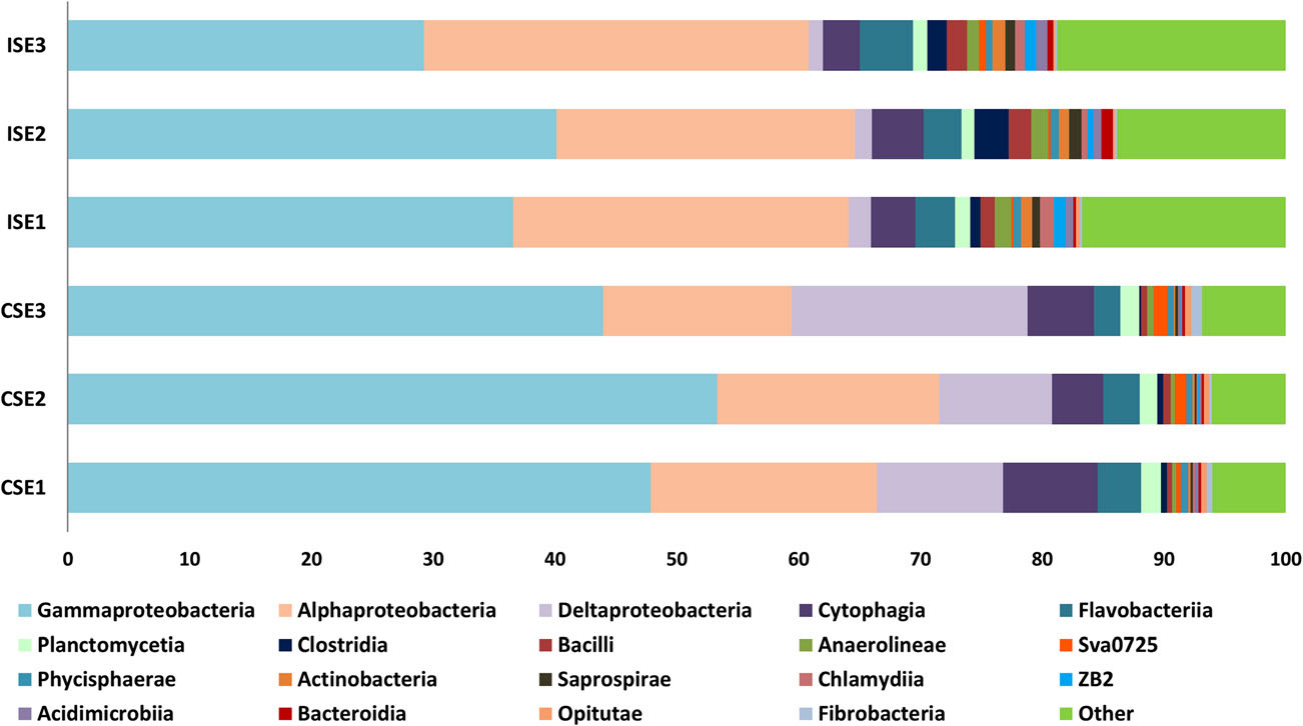
The structure of the microbial communities of endophytic bacteria associated with *S. europaea* from two salt-affected sites (I-ISE and C-CSE) revealed by IlluminaMiSeq 16S rRNA gene amplicon sequencing at the class level. Abundances lower than 5% were shown as “other”

Analysis of endophytic microbial structure at the class level indicated the presence of 106 different classes of bacteria in general. The higher abundance of endophytes was found for 19 classes namely Gammaproteobacteria, Alphaproteobacteria, Deltaproteobacteria, Cytophagia, Flavobacteria, Planctomycetia, Clostridia, Bacilli, Anaerolineae, Sva0725, Phycisphaerae, Actinobacteria, Saprospirae, Chlamydia, ZB2, Acidimicrobia, Bacteroidia, Opitutae, and Fibrobacteria at site C and I. The rest of identified and unassigned classes were presented as “other” (Fig. 5). The composition of the above-mentioned class of bacteria was closely related to the nature of the test site. Comparative analysis of the relative abundance of endophytic bacteria at the class level using linear discriminant analysis coupled with effect size (LEfSe) revealed 34 classes, which significantly distinguished the samples from the two investigated sites. The higher abundance of Holophagae, Cytophagia, Caldithrixae, Fibrobacteria, BB34, C6, OM190, Planctomycetia, 028H05-P-BN-P5, Deltaproteobacteria, Gammaproteobacteria, TA18, and Opitutae was noted for the endophytic bacterial population associated with *S. europaea* roots collected from the more saline site C (See Supplementary Material, Fig A). The bacteria classified to Acidobacteria Class 6, Acidimicrobiia, Actinobacteria, Nitriliruptoria, Thermoleophilia, Rhodothermi, Saprospirae, PRR-11, Chlamydia, OPB56, Anaerolineae, Thermomicrobia, Bacilli, Clostridia, Gemm_5, ZB2, WCHB1-64, Alphaproteobacteria, Spirochaetes, SJA-4, and TM7-1 were observed more often among endophytes from site I than site C (See Supplementary material, Fig B). Moreover, the occurrence of bacteria classified as Holophagae, Caldithrixae, 028H05-P-BN-P5, TA18, Deltaproteobacteria, C6, Sva0725, and Fibrobacteria was noted only at site C (See Supplementary Material, Figs A and B).

## Discussion

In this study, we assessed the effect of soil salinity and the physico-chemical parameters of the root zone soil that may be involved in structuring the diversity of endophytic bacteria in the roots of *S. europaea* growing at two saline sites. Using the Illumina sequencing approach, a higher density of the endophytic bacterial community associated with the roots of *S. europaea* from highly saline soils could be linked to the more complex and sophisticated relationships of bacteria inside the halophyte plant tissue.

### Bacterial communities in saline soils

A study by Borruso et al. (2014) on *Phragmites australis* (Cav.) Trin. ex Steud. rhizobacterial communities in a hypersaline pond and microbiota inhabiting bulk sediments showed a low variability of the microbial community structure among the sampled replicates. Similar results were seen in the structure of the endophytic bacterial populations in the three subsamples of *S. europaea* roots at each of our investigated plots. These observations suggest a strong effect of environmental conditions on the microbiome associated with plants. Significant differences between the two investigated sites with relevance to the density of the endophytic bacteria in *S. europaea* at the phylum level were observed using statistical analysis. These can be associated with differences in the level of the physico-chemical properties of root zone soil. Numerous scientific studies confirm the adequacy of this assumption. Zhang et al. (2016) found a positive correlation between the level of organic carbon, total nitrogen, and the number of *S. alterniflora* rhizobacteria belonging to the following phyla: Actinobacteria, BRC1, Firmicutes, and Gemmatimonadetes that align our observations (higher density of mentioned phyla was noted among endophytes from site I than at site C). Furthermore, the authors reported a negative correlation of the identified bacterial phyla with salinity, indicating the negative effect of this factor on the abundance of phylum Chloroflexi (Zhang et al. 2016). Canfora et al. (2014) also illustrated the relationship between organic carbon level and density of bacteria representing Chlorobi phylum. Site C (higher salinity) was characterized by a higher density of bacteria representing Acidobacteria, which, according to Foesel et al. (2014), have the ability to grow in an environment poor in nutrients. The above-mentioned data suggest that the distribution of bacteria may be determined by root zone soil physico-chemical parameters (such as organic carbon, total nitrogen content); however, salinity may play a crucial role in determining the density of endophytes representing individual phyla.

Our study also reveals that the higher levels of soil salinity did not decrease the composition of endophytic bacterial community diversity in roots of bacterial diversity. On the contrary, we noted a slightly higher abundance of OTUs at the more saline site C (about 113 dS m^−1^), compared to site I (about 64 dS m^−1^). Many studies (e.g., Ventosa and Arahal 2002; Guzman et al. 2008; Rueda-Puente et al. 2013; Yan et al. 2015) have shown that salt stress negatively affects populations of soil microorganisms; however, halotolerant and halophytic microorganisms can easily survive in salt stress conditions. This is also in line with our previously reported observations where we found a higher total bacterial biomass measured as the PLFA content in soil, rhizosphere, and roots of *S. europaea* at the more saline site (C) (Szymańska et al. 2016a). These findings can be a result of the different origins of salinity at the two test sites, which can influence the pattern of the bacterial community distribution in the soil. Bacteria representing Actinobacteria may exhibit lower tolerance to salt stress. This phylum exists more frequently in environments with lower levels of salinity (e.g., Herlemann et al. 2011; Dupont et al. 2014), while the higher amount of bacteria belonging to Verrucomicrobia was found in more saline environment (Yang et al. 2016; Herlemann et al. 2011), which is comparable to our results. The important role of time in an adaptation of microorganisms to salinity has been previously emphasized by Barin et al. (2015), who examined the effect of salinity on the structure of microbial communities in soils where lucerne, onion, and native *S. europaea* were cultivated. The time necessary for shaping the bacterial community at our investigated sites was longer in site C (natural salinity) which represents the naturally saline area and exists much longer as compared to the site I (anthropogenic salinity).

### Plants growing in saline soils

Halophytes can accumulate high amounts of salts in their tissues. Our results show an exceptionally higher content of not only Na^+^, Cl^−^ but also Mg^2+^, K^+^ in the roots and root zone soil of *S. europaea* from the more saline site C (113 dS m^−1^). These results are compatible with the existing knowledge on the role of Na^+^, K^+^, and Cl^−^ in maintaining cellular pressure in halophytes (Shabala 2013). Despite the lower levels of calcium present in the root zone soil at site C, the roots of this plant contain a higher level of this element. This may correspond to the significance of calcium in adaptation of plants to high salinity (Ben Amor et al. 2010), since this element plays an important role in the functioning of cell membranes, affecting their permeability and selectivity, and is involved in numerous metabolic processes in plant cells (Ben Amor et al. 2010). As in our research work, Yang et al. (2016) had also observed high concentrations of K^+^, Ca^2+^, Na^+^, and Mg^2+^ in sunflower roots grown in a saline environment. The authors justified the presence of high Ca^2+^ content in roots and its importance in preventing cellular damage (Yang et al. 2016). There are some reports considering the connection between high Ca^2+^ levels and an increase in antioxidant enzyme activity as well as on the reduction of lipid peroxidation in cell membranes (Ben Amor et al. 2010) under saline conditions.

### Bacterial microbiome vs. host specificity

Many researchers have observed high similarities in the populations of bacteria colonizing the same plant zones (e.g., leaf endophytes, leaf area, epiphytes) that came from distant sites (Mora-Ruiz et al. 2015). The OTUs obtained for bacterial endophytes of *S. europaea* from the two test sites in this study belonged to 36 phyla with 12 dominating in the decreasing order from Proteobacteria, Bacteriodetes, Firmicutes, Planctomycetes, Acidobacteria, and Actinobacteria, Chloroflexi, Verrucomicrobia, OD1, Chlamydiae, TM7 as well as Fibrobacteres. Shi et al. (2015) examined the diversity of bacteria associated with two halophytes: *Salicornia europaea* and *Sueada aralocaspica*. They revealed a lower number of different phyla (25 in total) with domination of bacteria representing Proteobacteria (41.61–99.26%; average, 43.30%), Firmicutes (0–7.19%; average, 1.15%), Bacteroidetes (0–1.64%; average, 0.44%), and Actinobacteria (0–0.46%; average, 0.24%). However, their study was conducted in saline desert soils, which is less favorable for microbial colonization (Shi et al. 2015). Moreover, Zhao et al. (2016) analyzed the biodiversity of bacterial endophytes in roots of *S. europaea* using the metagenomic approach that noted an increased frequency of some phyla: Proteobacteria (95.3%) > Bacteroidetes (2.6%) > Actinobacteria (0.9%) > Firmicutes (0.6%), which is in accordance with our results. They also confirm high species specificity of bacterial endophytes of *S. europaea*, but with differences in their abundance, which may correspond to the specific soil properties. Mora-Ruiz et al. (2015) reported the diversity of endophytic and epiphytic bacteria associated with members of the subfamily *Salicornioideae* originating from five different research sites located in Spain and Chile, which concluded that the host plant genotype determines the taxonomic diversity of microorganisms. To summarize, the obtained results and observations of other scientists confirm that the host halophyte species significantly determine the endophytic bacterial community diversity.

The results from this research and from previous reports (e.g., Mukhtar et al. 2016; Ma and Gong 2013; Yang et al. 2016) have stated that salinity may significantly affect the structure of different types of microorganisms living both in strong associations within the plants as well as in the root zone soil. For instance, the analysis of the distribution of bacteria in rhizosphere, rhizoplane, and histoplane of para grass (*Urochloa mutica*), a salt tolerant plant species growing in a saline environment, showed predominance of bacteria belonging to Proteobacteria (16.67%), Firmicutes (16.67%), Acidobacteria (12.5%), Bacteroidetes (4.2%), Cyanobacteria (4.2%) as well as Actinobacteria, Choroflexi, Gemmatonadetes, and Planctomycetes (2.1% each type) (Mukhtar et al. 2016). Comparable to our observations, the domination of bacteria belonging to phylum Proteobacteria, Acidobacteria, Bacteroidetes, and Actinobacteria among root zone soil bacteria of *Helianthus tuberosus* L. was reported by Yang et al. (2016). Ma and Gong (2013) noted bacteria belonging to Proteobacteria (44.9%), Actinobacteria (12.3%), Firmicutes (10.4%), Acidobacteria (9.0%), Bacteroidetes (6.8%), and Chloroflexi (5.9%) phyla in saline soil. Bacterial phyla observed in our work were also reported by Canfora et al. (2014) in saline soils originating from nine research sites in Sicily (Italy). The authors identified bacteria representing phyla Proteobacteria, Actinobacteria, Acidobacteria, and Verrucomicrobia, and a lower occurrence of bacteria belonging to Bacteroidetes, Chloroflexi, Chlorobi, and Gemmatomonadates was reported (Canfora et al. 2014). The dominance of bacteria representing phyla Proteobacteria, Bacteroidetes, and Actinobacteria was also observed among endophytes originating from non-saturated environments (Akinsanya et al. 2015; Pei et al. 2017), which indicates a high adaptation of these bacteria to salt stress conditions. The two test sites examined in this study have indicated a very similar distribution of Bacteroidetes (C 9.2%, I 8.8%) and Planctomycetes (C 2.3%, I 1.9%). These observations may suggest that the presence of certain phyla of bacteria in a saline environment is constant. This was also reported by Zhang et al. (2016), wherein the lack of differences in the abundance of *Spartina alterniflora* (halophyte) endophytes belonging to Bacteroidetes in most of their researched sites (10 out of 12) was seen, although these sites differed in salinity and other soil properties.

The metagenomic analysis showed phylum Proteobacteria to be dominant at the two sites, but a significantly higher density was noted in site C (78.96%) being more saline than the site I (64.85%). Observations of many scientists about Proteobacteria are not always in accordance with results obtained in this work (e.g., the statement of negative influence of salinity on their numbers (Yang et al. 2016), or a positive correlation between the abundance of Proteobacteria and the high levels of organic carbon and total nitrogen (Pii et al. 2016)). For this reason, an additional bioinformatics analysis on a lower systematic level (classes) was carried out in the present work, since Proteobacteria is represented by differential classes of bacteria. At the class level distribution, a significant number of OTUs belonged to Deltaproteobacteria and Gammaproteobacteria from the site (C). A high density of bacteria representing the above classes was also found in marine sediments (Wang et al. 2009), thus depicting an adaptation of this group of bacteria to environments characterized by high salinity. Our assumption has been confirmed by other researchers in the case of assessing the diversity of bacteria present in the Baltic Sea, which revealed the adaptation of Gammaproteobacteria to high salinity (Dupont et al. 2014; Herlemann et al. 2011). In another paper on the diversity of lucerne root endophytes (*Medicago truncatula*), a higher abundance of Gammaproteobacteria was observed in saline soils than among the control samples (without salt) (Yaish et al. 2015). Hence, the above evidence confirms the important role of salinity in determining the occurrence of Gammaproteobacteria and Deltaproteobacteria.

## Conclusions

In conclusion, we have revealed that the roots of *S. europaea* are naturally associated with diverse endophytic bacterial communities in which the distribution is affected by environmental factors (salinity) at naturally and anthropogenically saline test sites in Poland demonstrated in this study. Contrary to our predictions, salinity did not adversely affect the biodiversity of *S. europaea* endophytes. Moreover, the results revealed a slightly higher number of endophytic bacterial OTUs at the much older and more saline test site. Our analyses have shown that endophytes representing phyla Proteobacteria and Bacteroidetes predominate in saline environments regardless of the level of salinity in the root zone soil and plant roots. In addition, to the above-mentioned phyla, the representatives of Planctomycetes and Acidobacteria were more commonly found in a more saline site, while Firmicutes, Proteobacteria, and Chloroflexi occurred more often in a less saline environment. The most halotolerant and halophytic bacteria were found to belong to the following phyla: Acidobacteria, Caldithrix, Fibrobacteres, Proteobacteria (primarily Deltaproteobacteria), and Verrucomicrobia. We observed a significantly low density of endophytic bacteria representing 12 phyla (Actinobacteria, BRC1, Chlamydiae, Chlorobi, Chloroflexi, Firmicutes, Gemmatimonadetes, OD1, OP11, Spirochates, TM6, and TM7) in the more saline test site, which may indicate that endophytes belonging to these phyla are characterized as being inferior to adaptation in environments with high level of salt stress.

## Electronic supplementary material

ESM 1 (TIF 2021 kb)

High Resolution Image (PNG 596 kb)

ESM 2 (TIF 1799 kb)

High Resolution Image (PNG 474 kb)
