# Supplementary figures and images for: Bacterial microbiome of root-associated endophytes of Salicornia europaea in correspondence to different levels of salinity
Source: Environ Sci Pollut Res Int. 2018 Jun 27;25(25):25420–31. doi: 10.1007/s11356-018-2530-0 (PMC6133108; doi:10.1007/s11356-018-2530-0)

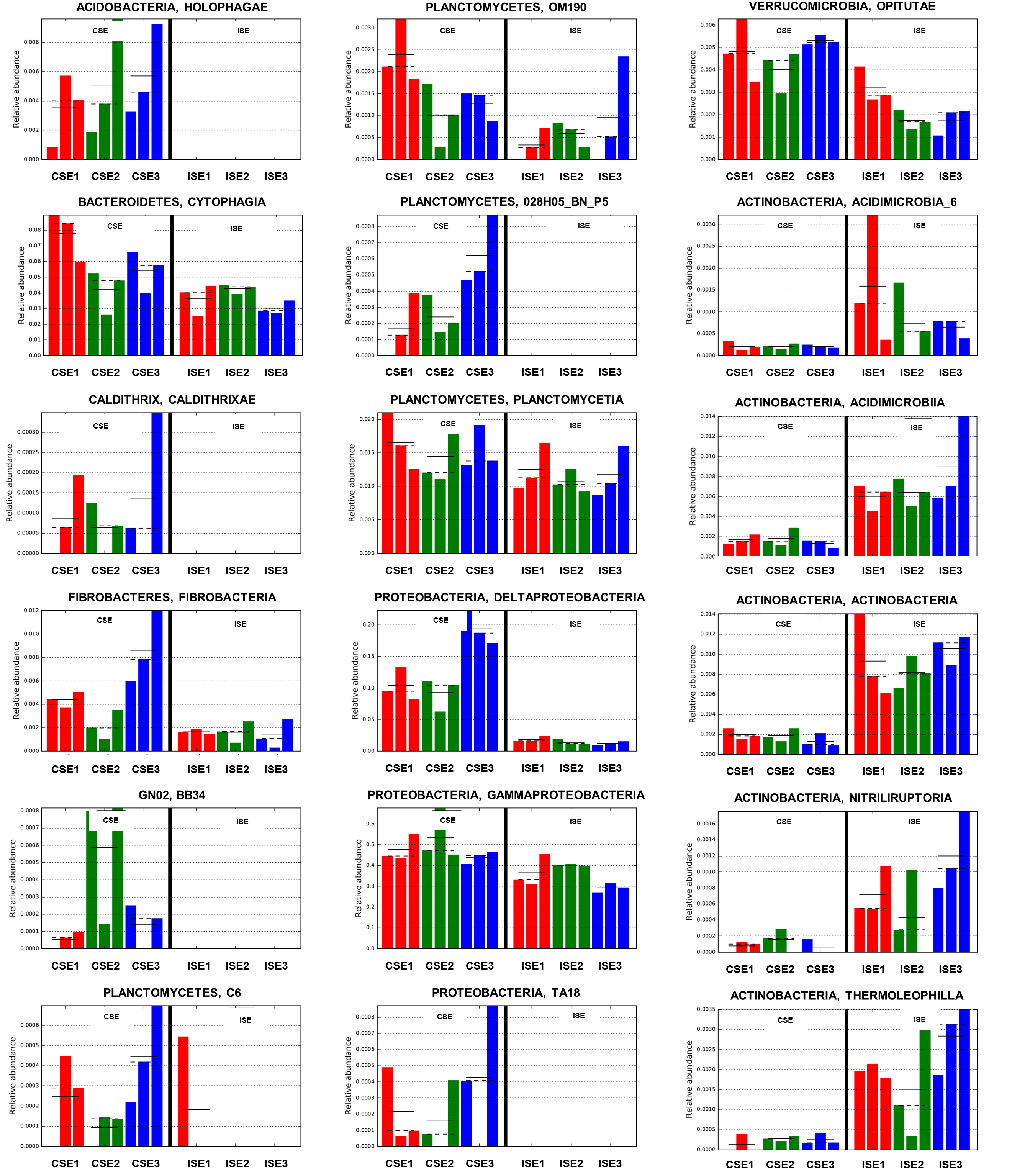

Supplement: Supplementary file 1 — (TIF 2021 kb) [file 11356_2018_2530_MOESM1_ESM.tif]

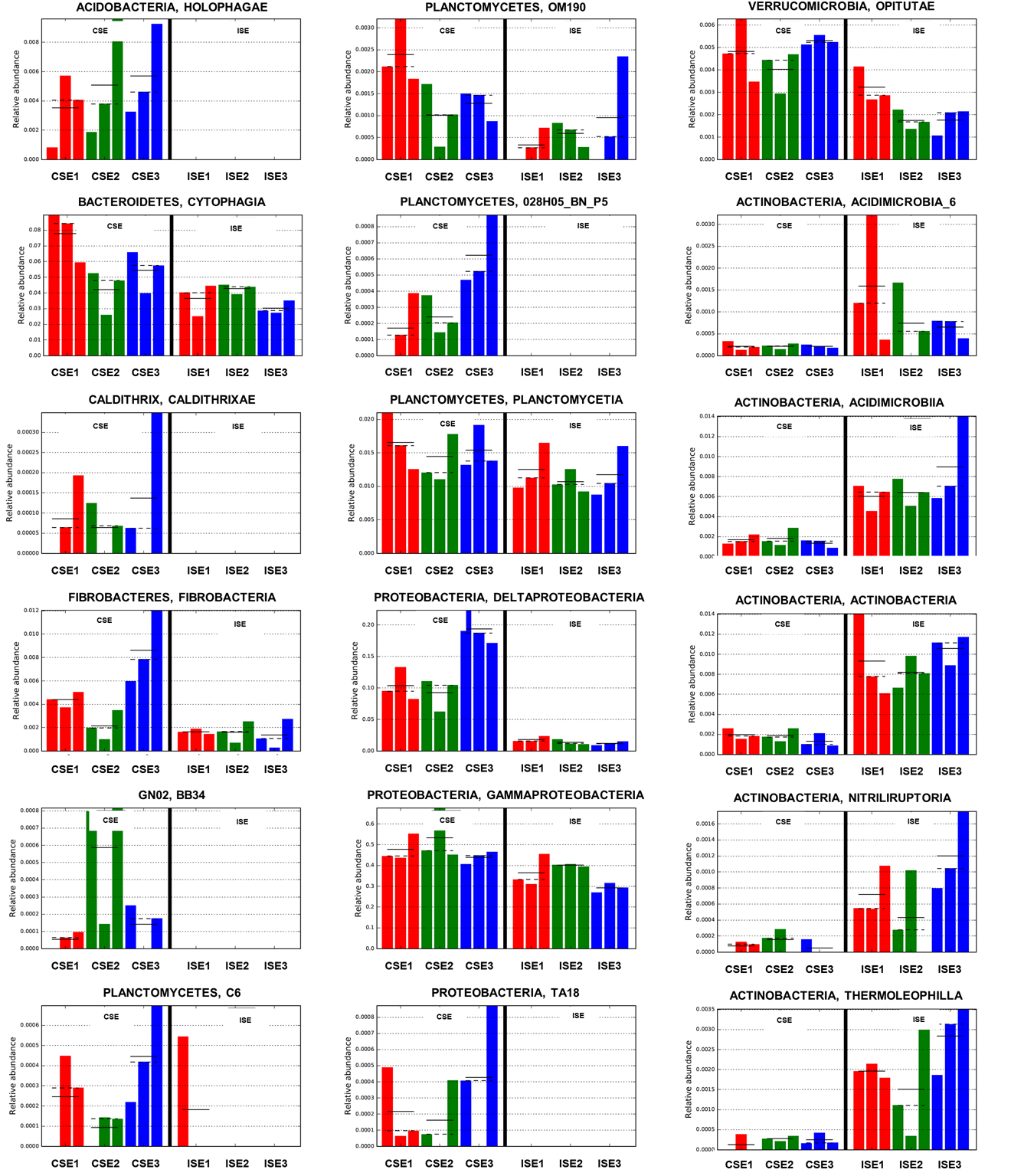

Supplement: Supplementary file 2 — High Resolution Image (PNG 596 kb) [file 11356_2018_2530_Fig6_ESM.png]

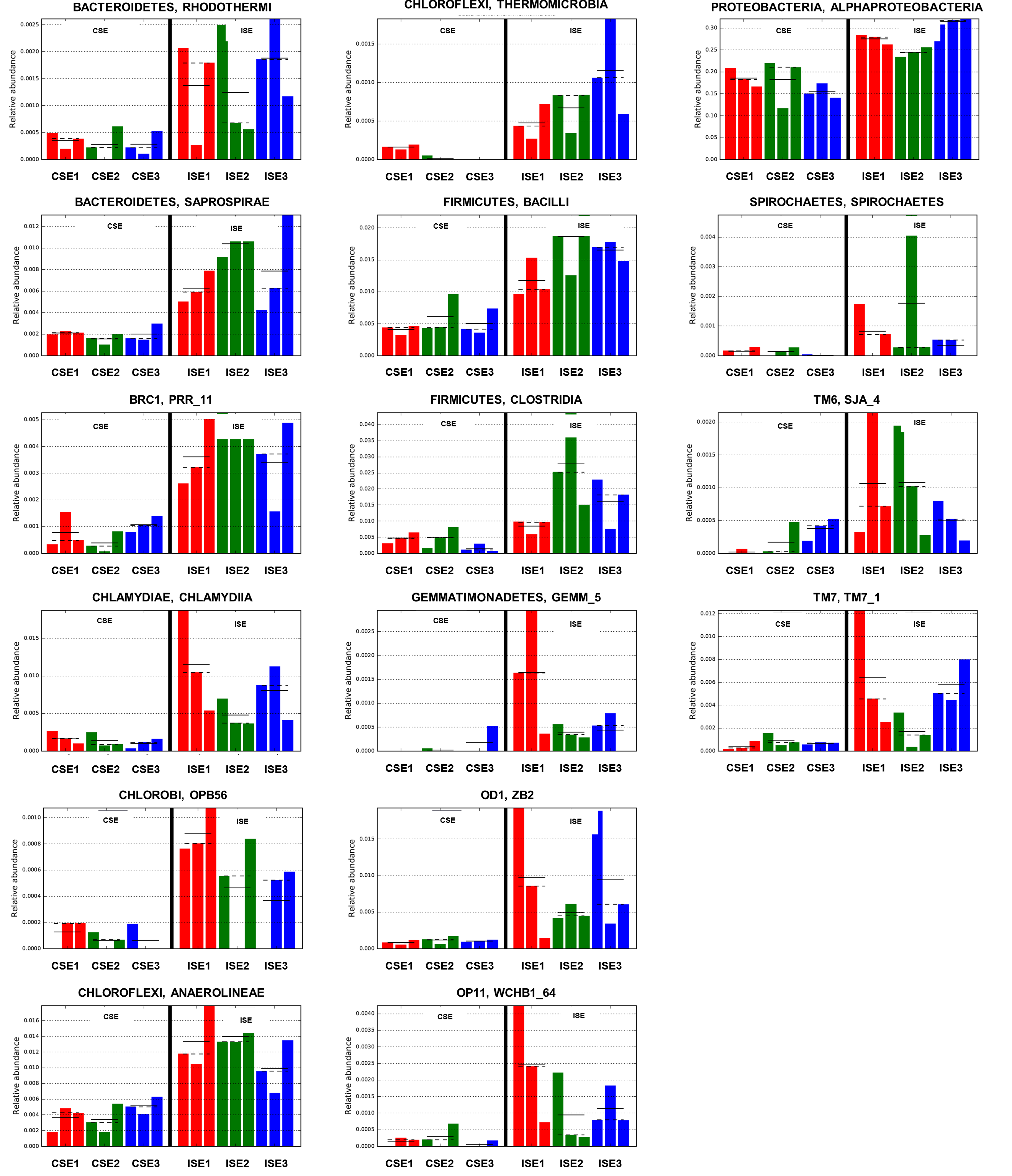

Supplement: Supplementary file 3 — (TIF 1799 kb) [file 11356_2018_2530_MOESM2_ESM.tif]

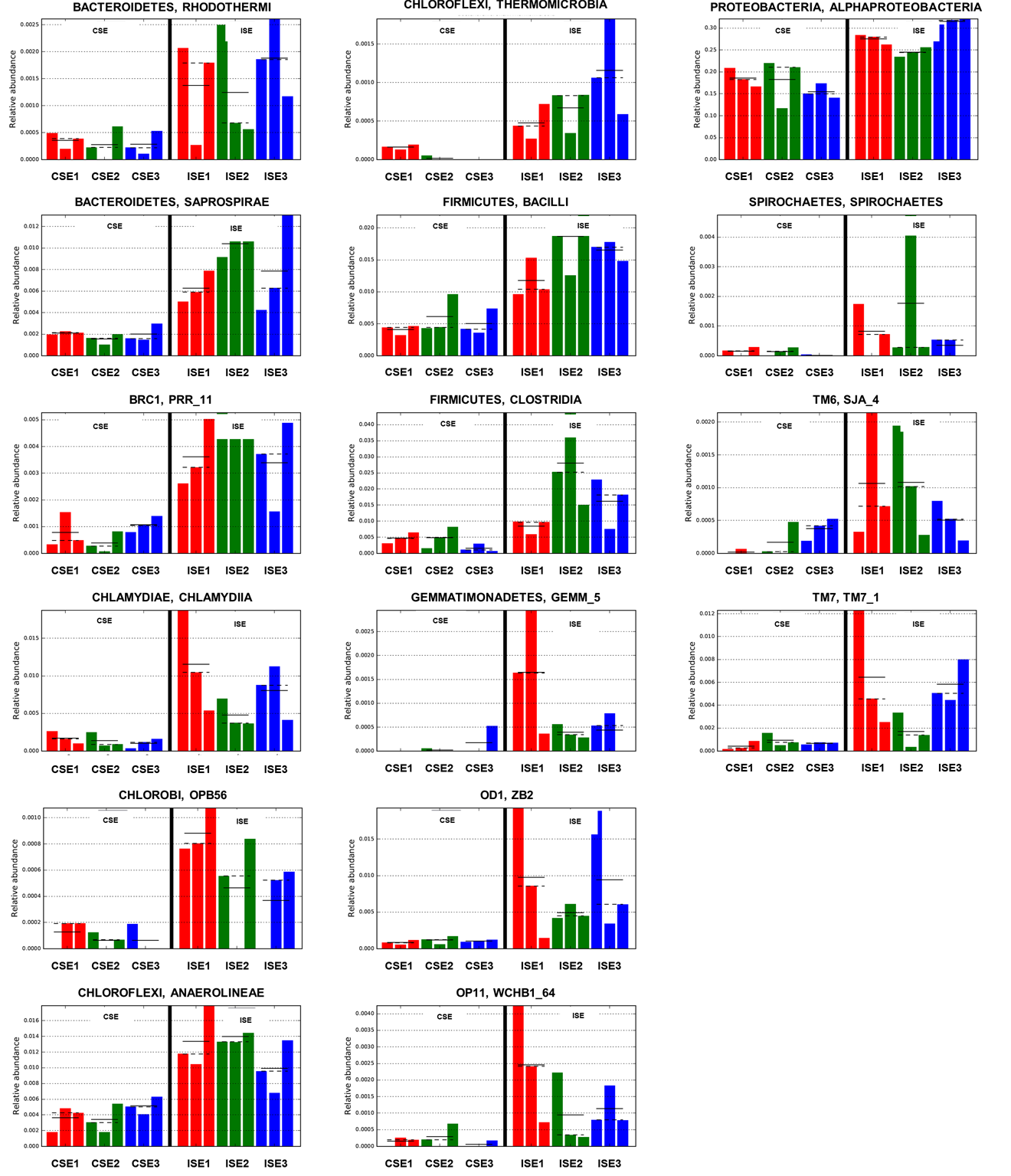

Supplement: Supplementary file 4 — High Resolution Image (PNG 474 kb) [file 11356_2018_2530_Fig7_ESM.png]
